# Molecular pathways and immune microenvironment regulation in stem cell therapy for thin endometrium: a comprehensive narrative review

**DOI:** 10.3389/fimmu.2026.1804457

**Published:** 2026-04-23

**Authors:** Xiaodan Yin, Mengyuan Li, Qian Han, Siqi Guan, Junqin He

**Affiliations:** Department of Traditional Chinese Medicine (TCM), Beijing Obstetrics and Gynecology Hospital, Capital Medical University, Beijing Maternal and Child Health Care Hospital, Beijing, China

**Keywords:** biomaterials, immune microenvironment, molecular pathways, stem cell therapy, thin endometrium

## Abstract

A thin endometrium is a significant contributor to female infertility and adverse pregnancy outcomes and remains a persistent challenge in reproductive medicine. Stem cell therapy, when integrated with biomaterials and tissue engineering, has emerged as a promising approach for thin endometrial repair. This review systematically summarizes the critical molecular pathways involved in stem cell-based therapy for thin endometrium and explores the regulatory mechanisms within the immune microenvironment. This review focuses on the role of stem cells and their exosomes in promoting angiogenesis, exerting antifibrotic effects, and modulating immune responses. The integration of advanced biomaterials is also discussed, highlighting their fundamental roles in optimizing the stem cell transplantation microenvironment and facilitating endometrial regeneration. This review integrates recent insights into the molecular mechanisms and immune regulation to provide a theoretical framework and clinical guidance for precise therapies targeting thin endometrium.

## 1 Introduction

**Figure 1:**
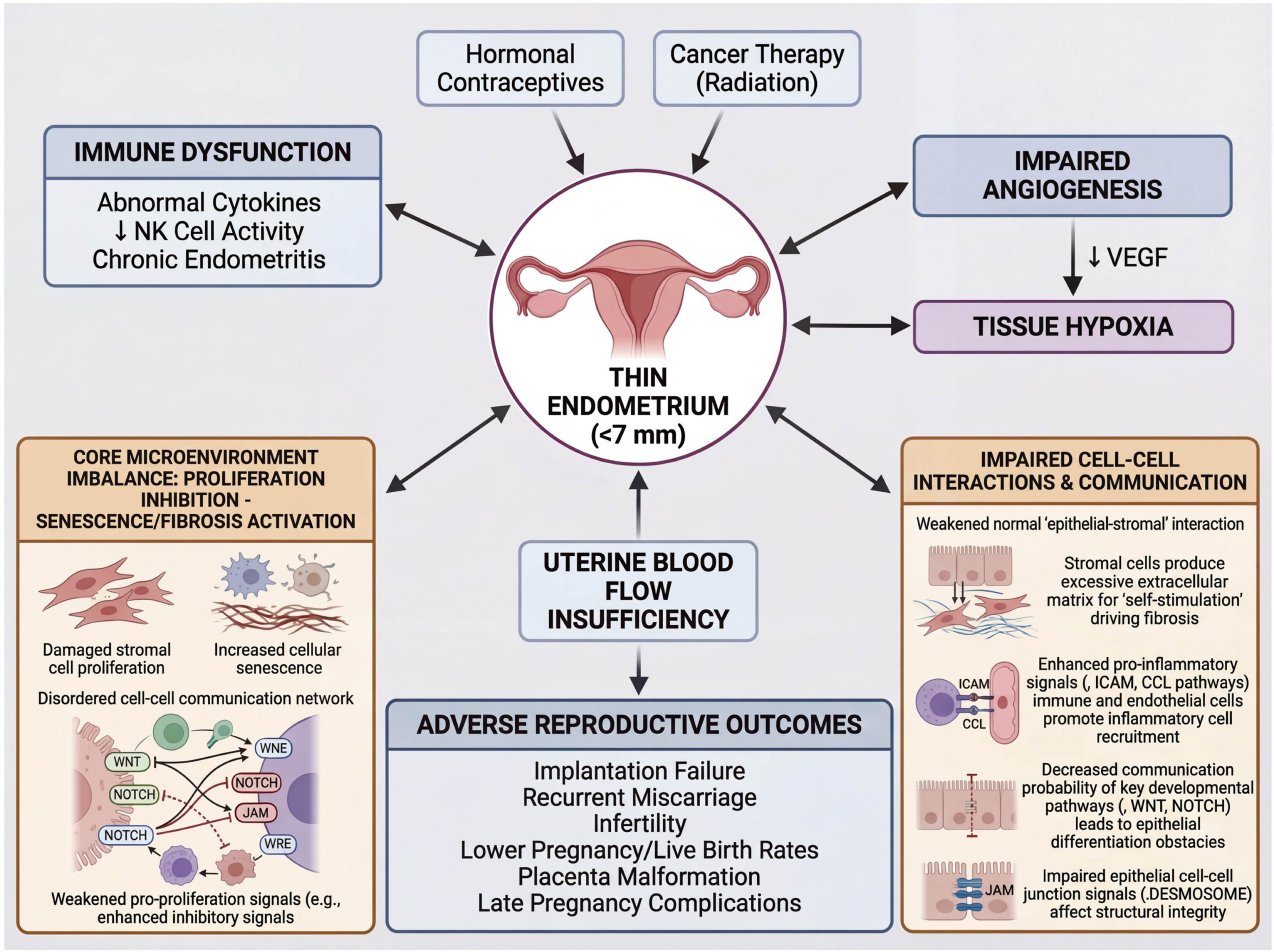
Pathophysiological mechanism of thin endometrium. Created by Biorender https://BioRender.com/h2idihg.

A thin endometrium (TE) is increasingly acknowledged as a key contributor to female infertility and recurrent implantation failure. TE, characterized by insufficient endometrial thickness, presents substantial obstacles to successful embryo implantation and subsequent pregnancy, making this condition a critical focus in reproductive medicine. Its pathophysiology is a complex, multifactorial process involving impaired angiogenesis, tissue hypoxia, immune abnormalities, and epigenetic changes. The latest research also found that the core pathology of TE is the microenvironmental imbalance of “proliferation inhibition-senescence/fibrosis activation”, which is manifested by impaired stromal cell proliferation, aggravated cell senescence and excessive collagen deposition. Intercellular communication networks are significantly disrupted; key pro-proliferative signals are weakened while inhibitory signals are enhanced, collectively leading to compromised endometrial regenerative capacity (1). It was also found that the normal “epithelial-stromal” interaction of the TE is weakened, and the stromal cells “self-stimulate” by producing excess extracellular matrix, driving fibrosis. The pro-inflammatory signals (such as ICAM and CCL pathways) between immune cells and endothelial cells are enhanced, which promotes inflammatory cell recruitment. The signaling activity of key developmental pathways (e.g., WNT, NOTCH) decreases, resulting in defects in epithelial differentiation. Epithelial intercellular junctional components (e.g., desmosomes, JAMs) are impaired, compromising structural integrity (2)(**Figure 1**).

Stem cell therapy has emerged as a promising strategy for managing the complexities of TE, as it leverages cellular regenerative and immunomodulatory properties to provide a comprehensive approach for improving endometrial repair and function.

Recent studies have highlighted the significance of different stem cell types in facilitating endometrial regeneration. Mesenchymal stem cells (MSCs), derived from sources such as the human placenta, umbilical cord, and menstrual blood, are notable for their ability to differentiate into endometrial cells and secrete growth factors that promote tissue repair. Research indicates that Encapsulating human placenta-derived MSCs in hyaluronic acid hydrogels notably increases endometrial thickness and boosts embryo implantation rates in animal models of endometrial injury (3). Umbilical cord-derived MSCs (UC-MSCs) can enhance endometrial function by modulating the extracellular matrix and inflammatory responses essential for endometrial health (4).

The mechanisms underlying the regenerative potential of MSCs in TE are multifactorial. MSCs can differentiate into multiple cell types and exert paracrine effects that enhance cellular proliferation, angiogenesis, and tissue remodeling. MSCs activate paracrine signaling pathways such as the JNK/Erk1/2-Stat3-VEGF cascade, thereby enhancing endometrial stromal cell proliferation and migration (5). Additionally, combining biomaterials, such as collagen scaffolds or hydrogels, with stem cell therapy has been investigated to enhance cell retention and therapeutic efficacy. These biomaterials provide a supportive microenvironment that enhances stem cell viability and functionality, thereby increasing the efficacy of regenerative therapies (6).

Despite the promising results from preclinical studies, challenges remain in translating these therapies into clinical practice. Injectable hydrogels capable of encapsulating stem cells and gradually releasing bioactive factors have been investigated to enhance endometrial regeneration (7). Identification of the molecular pathways involved in endometrial repair, including the Wnt signaling pathway and its interaction with long non-coding RNAs (lncRNAs), presents novel therapeutic targets (8).

This review aims to provide a comprehensive narrative review and critical evaluation of the current understanding of how stem cell-based therapies influence molecular pathways and the immune microenvironment in the treatment of TE. The review covers the mechanisms by which stem cells modulate key signaling pathways and regulate immune cells to promote endometrial regeneration and improve receptivity.

A literature search was conducted using databases such as PubMed, Google Scholar, and Web of Science, covering studies published between 2021 and 2026. Keywords included “Thin endometrium”, “Stem cell therapy”, “Molecular pathways”, “Immune microenvironment”, and “Biomaterials”. Both preclinical and clinical studies were included. The selection process involved screening titles and abstracts for relevance, followed by a full-text review that included studies focusing on mechanistic insights and therapeutic outcomes while excluding unrelated or low-quality reports.

## 2 Molecular pathway mechanism of stem cell therapy for TE(**Figure 2**)

**Figure 2:**
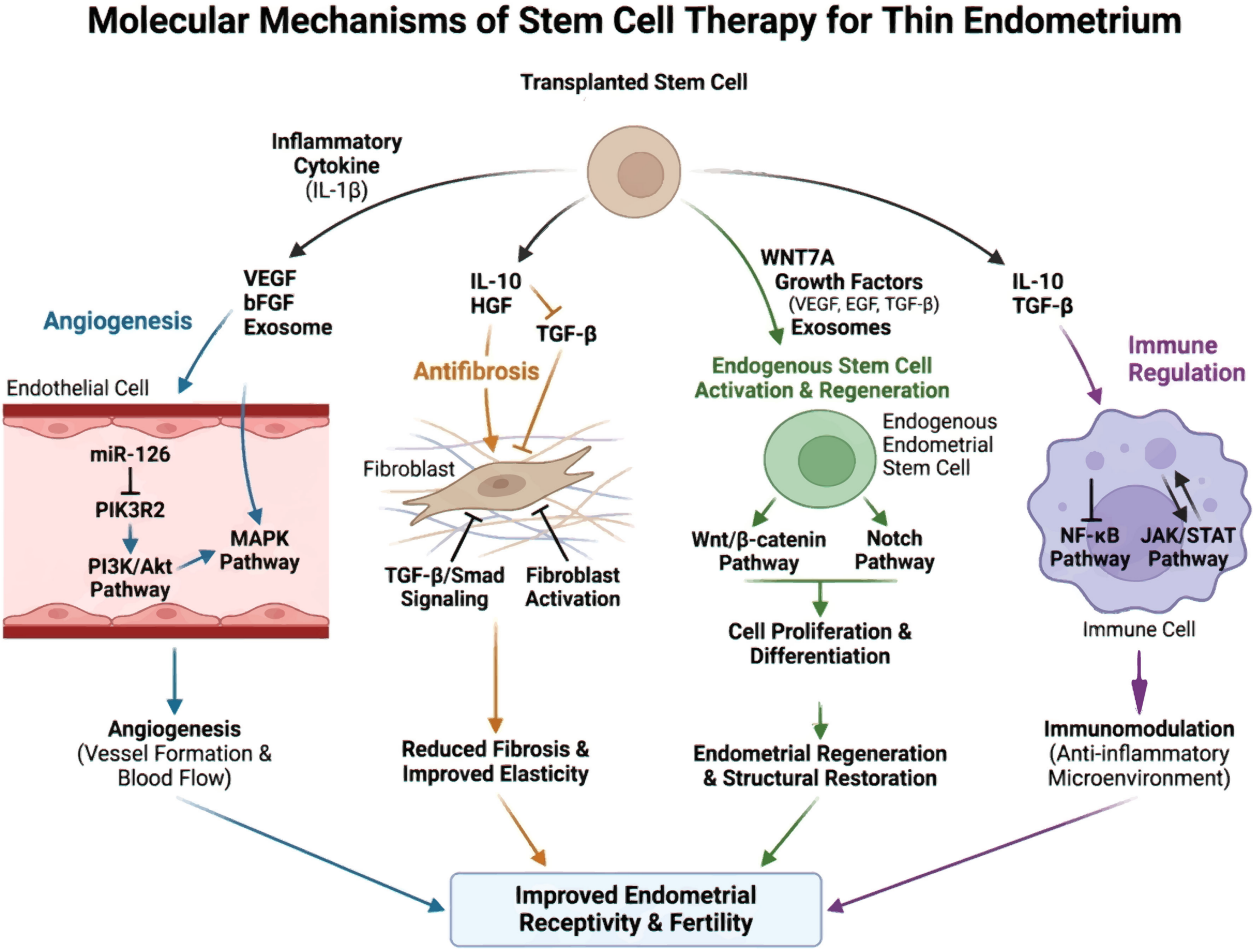
Molecular pathway mechanism of stem cell therapy for TE. Created by Biorender https://BioRender.com/h2idihg.

### 2.1 Angiogenesis-related signaling pathways

Angiogenesis, defined as the formation of new blood vessels from the pre-existing vasculature, plays a critical role in tissue repair and regeneration, particularly in stem cell-based therapies for tissue engineering. These angiogenic signaling pathways are crucial for endothelial cell proliferation, migration, and capillary structure formation, which are processes that improve blood supply and nutrient delivery to the endometrial tissue. Activation of the PI3K/Akt pathway is associated with elevated expression of angiogenic markers, enhanced endothelial cell survival and proliferation, and improved endometrial vascularization (9). Additionally, the MAPK signaling pathway regulates endothelial cell functions such as migration and tube formation, which are crucial for the development of a functional vascular network (10).

In cases of TE, in which the vascular supply is often compromised, stimulation of angiogenesis is crucial for restoring tissue integrity and function. Stem cells derived from bone marrow or adipose tissue promote angiogenesis by releasing exosomes containing microRNAs (miRNAs) that modulate angiogenesis-related gene expression. Exosomes derived from bone marrow mesenchymal stem cells (BMSCs) and enriched with miR-126 markedly promote angiogenesis through the inhibition of phosphoinositide 3-kinase regulatory subunit 2 (PIK3R2), which subsequently activates the PI3K/Akt signaling pathway (11).

Moreover, the interplay between stem cells and angiogenic signaling pathways is enhanced by the local inflammatory microenvironment. Interleukin-1 beta (IL-1β) primes stem cells through upregulation of essential angiogenic factors, such as basic fibroblast growth factor (bFGF) and vascular endothelial growth factor (VEGF), thereby enhancing their angiogenic potential (12).

In conclusion, stem cells enhance endometrial vascularization through the activation of angiogenesis-related signaling pathways and the secretion of proangiogenic factors and exosomal miRNAs. This angiogenic process enhances blood circulation and nutrient delivery, thereby fostering an environment favorable for tissue regeneration. Understanding these molecular mechanisms may facilitate the development of targeted therapies that enhance endometrial health and fertility, particularly in patients with TE.

### 2.2 Antifibrotic mechanisms and signal regulation

MSCs represent a promising therapeutic strategy for addressing tissue engineering challenges, largely because of their antifibrotic capabilities. A key mechanism by which MSCs mediate these antifibrotic effects involves the suppression of the transforming growth factor-beta (TGF-β)/Smad signaling pathway, a central regulator in fibrotic processes. Research indicates that MSCs secrete factors that inhibit TGF-β activity, leading to decreased collagen buildup and improved endometrial elasticity (13). This restoration of elasticity is crucial for endometrial function, as a fibrotic endometrium can severely impair implantation and overall fertility. MSCs modulate the TGF-β/Smad pathway, thereby preventing fibrosis and promoting a healthier endometrial environment conducive to successful embryo implantation.

MSCs not only inhibit TGF-β signaling but also release anti-inflammatory cytokines and growth factors crucial for diminishing fibrosis-associated cell activity. The release of factors such as IL-10 and Hepatocyte Growth Factor (HGF) can suppress inflammation and prevent fibroblast activation (14). Moreover, the secretome derived from MSCs enhances the proliferation and migration of endothelial cells, which are essential processes for angiogenesis and the reconstruction of normal endometrial architecture (15). By establishing a more conducive microenvironment, MSCs can support the repair of impaired tissues and stimulate endometrial regeneration, ultimately enhancing the endometrial receptivity for successful embryo implantation.

Reducing fibrosis helps restore the structural integrity of the endometrium and improve its functional capacity. The reduction in fibrotic tissue allows improved vascularization and nutrient exchange, both of which are essential for embryonic development (16). The interplay between MSCs and the local immune system is crucial. This immunomodulatory effect of MSC-mediated immune modulation is particularly beneficial in the endometrium, where a balanced immune response is necessary for successful implantation and pregnancy (17).

In summary, MSCs reverse endometrial fibrosis and restore endometrial function by inhibiting the TGF-β/Smad pathway and secreting anti-inflammatory factors. The ability of MSCs to create a regenerative microenvironment not only facilitates the repair of damaged tissues but also addresses the underlying pathophysiological conditions that contribute to infertility, making them a valuable therapeutic option in reproductive medicine.

### 2.3 Regulation of cell proliferation and differentiation

Stem cells are essential for endometrial regeneration, particularly in addressing TE, which is a major challenge in reproductive medicine. The Wnt/β-catenin and Notch signaling pathways are known to enhance stem cell proliferation and differentiation into endometrial epithelial and stromal cells. The Wnt/β-catenin signaling pathway is crucial for cellular processes such as proliferation, differentiation, and tissue homeostasis. Wnt signaling aids in the transformation of stem cells into functional endometrial cells, supporting the regeneration of the endometrial lining that is essential for implantation and pregnancy (18). Activation of Notch signaling in stem cells is associated with enhanced proliferation and formation of a differentiated endometrial phenotype, which is crucial for maintaining a healthy endometrial environment (19).

Disruption of these signaling pathways can result in insufficient endometrial regeneration, a phenomenon commonly associated with conditions such as TE and Asherman syndrome (20). Aberrant Wnt signaling has been implicated in diminished cellular proliferation and compromised endometrial cell differentiation, ultimately leading to reduced endometrial thickness and functional impairment (21).

Moreover, the interplay between these signaling pathways and the surrounding microenvironment is crucial for optimal stem cell function. The endometrial niche’s extracellular matrix and growth factors of the endometrial niche can significantly affect stem cell behavior by activating the Wnt and Notch pathways (20). Research indicates that extracellular matrix (ECM) components can activate pathways that support both cell proliferation and the preservation of stemness in endometrial stem cells (22). This finding highlights the importance of a well-regulated microenvironment that supports the signaling mechanisms necessary for effective endometrial regeneration.

The manipulation of these pathways may advance reproductive medicine and provide new hope for individuals with endometrial dysfunction-induced infertility. Future research should focus on elucidating intricate signaling pathway networks and their interplay with the endometrial microenvironment to improve stem cell-based approaches for endometrial regeneration (23).

### 2.4 Molecular actions of stem cell-derived exosomes

Stem cell-derived exosomes are small extracellular vesicles that play a pivotal role in intercellular communication and have attracted substantial interest in regenerative medicine. For example, lncRNAs encapsulated within exosomes can interact with target mRNAs and miRNAs, thereby regulating signaling pathways involved in cellular responses (24). Exosomes facilitate cellular communication by delivering molecular content to recipient cells, thereby enabling coordinated responses to injury or disease and offering a promising therapeutic approach for tissue restoration and regeneration.

Exosome-mediated signaling plays a pivotal role in intercellular communication and is vital for tissue regeneration, particularly in the endometrium. Furthermore, stem cell-derived exosomes support angiogenesis and ECM remodeling, both of which are fundamental mechanisms underlying effective wound healing (25). These properties highlight the therapeutic potential of exosomes in regenerative medicine, especially under conditions characterized by impaired healing such as chronic wounds and fibrotic disorders.

Exosomes enable clinicians to harness the regenerative capacity of stem cells while avoiding the challenges associated with live-cell therapies. This approach has been validated in various preclinical models in which exosomes have been shown to reduce inflammation, promote tissue repair, and enhance functional recovery in damaged tissues (26). Current research has actively explored exosome engineering to improve targeting and therapeutic effectiveness, with promising results for the efficient delivery of therapeutic agents to specific tissues or cells (27). This novel approach has the potential to transform the treatment of numerous diseases including reproductive system disorders by offering a safer and more effective tissue regeneration method.

In conclusion, stem cell-derived exosomes offer a promising approach to regenerative medicine because they modulate cellular behavior and facilitate tissue repair through the delivery of bioactive molecules. Ongoing research on the mechanisms of exosomes is expected to broaden their clinical applications, leading to innovative and safer treatments that more effectively harness the regenerative potential of stem cells.

### 2.5 Interaction between stem cells and endometrial stem cells

The interaction between transplanted and endogenous endometrial stem cells plays a pivotal role in enhancing the self-repair capacity of the endometrium. This synergistic interplay fosters tissue regeneration and remodeling, which are critical for successful embryo implantation and the maintenance of reproductive health. Stem cells, especially those from the menstrual blood or other sources, can migrate to injury sites and interact with local endometrial stem cells, thereby fostering a repair-friendly microenvironment. Studies have demonstrated that UC-MSCs secrete WNT7A, a factor that activates endometrial epithelial stem cells and facilitates regeneration of the endometrial epithelial layer (28). Stem cell-derived exosomes carry growth factors and miRNAs that support the proliferation and differentiation of resident endometrial stem cells, thereby contributing to tissue repair (29). This synergistic interaction strengthens both the structural stability and functional performance of the endometrium, ultimately resulting in enhanced fertility.

Additionally, transplanted stem cells release growth factors and cytokines that markedly boost the function and expansion of endometrial stem cells. The paracrine actions of these stem cells include the secretion of diverse signaling molecules capable of influencing the behavior of adjacent cells. This effect is especially noticeable in endometrial repair, where factors such as VEGF, epidermal growth factor(EGF), and TGF-β are produced to activate the local stem cell pool. These molecules promote formation of new blood vessels, enhance cell growth, and regulate immune responses, all of which are vital for successful tissue regeneration (30). The interplay between these secreted factors and the intrinsic signaling pathways of endometrial stem cells leads to a robust regenerative response.

**Figure 3:**
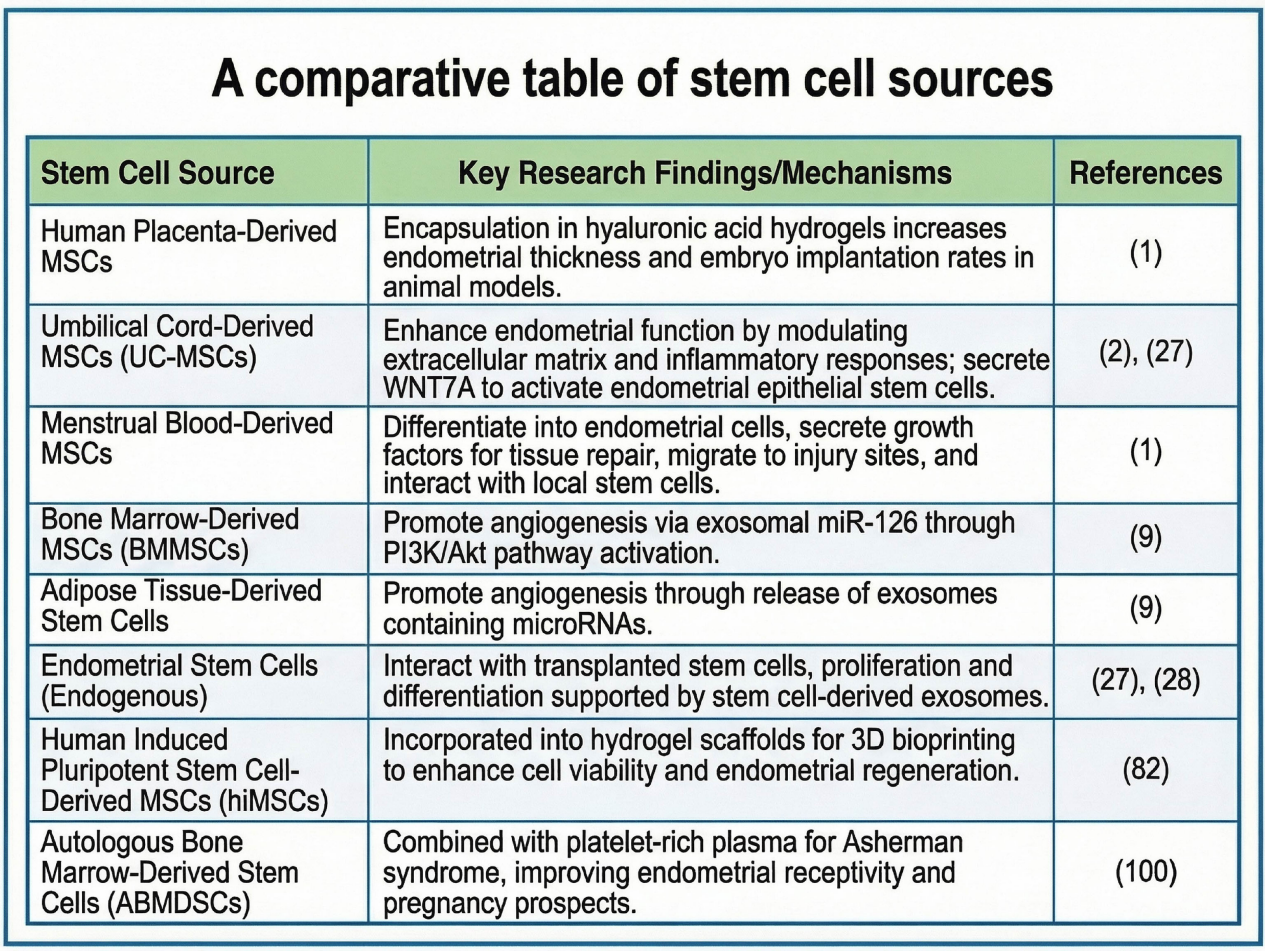
A comparative table of stem cell sources. Created by Biorender https://BioRender.com/h2idihg.

The interaction between the transplanted and endometrial stem cells underpins the sustained effectiveness of stem cell therapies for endometrial dysfunction. For instance, the identification of specific growth factors that enhance endometrial receptivity could lead to innovative treatments for conditions such as intrauterine adhesions and TE, which are major contributors to infertility (31). Stem cell-derived exosomes offer a promising therapeutic approach for enhancing endometrial repair while avoiding the complications associated with direct cell transplantation (32). A comparative table of stem cell sources is depicted in **Figure 3**.

### 2.6 Stem Cell-mediated immune regulatory pathways

MSCs play a pivotal role in modulating immune responses through diverse molecular mechanisms. These signaling molecules are essential for regulating local immune activity because they suppress the activation and expansion of proinflammatory immune cells, thereby alleviating inflammatory conditions. IL-10 reduces the expression of proinflammatory cytokines and facilitates the development of regulatory T cells, strengthening the immunosuppressive milieu. Meanwhile, TGF-β not only restrains T cell activation but also encourages MSCs to adopt a phenotype with enhanced immunosuppressive properties. Stem cells are vital for preserving tissue balance and curbing excessive inflammation, particularly during transplantation and tissue regeneration, because they secrete immunosuppressive mediators and perform fine immune cell functions (33).

Stem cells influence immune responses through multiple mechanisms that extend beyond cytokine secretion, including the regulation of key signaling pathways. The NF-κB pathway is essential for immune regulation, primarily by regulating the expression of proinflammatory cytokines. Stem cells can attenuate the NF-κB pathway through the release of soluble factors that suppress pathway activation, which leads to decreased cytokine production and fosters an anti-inflammatory environment. Similarly, the JAK/STAT pathway mediates the signaling of various cytokines, including those secreted by immune cells. Stem cells modulate the JAK/STAT pathway, reduce proinflammatory cytokine levels, enhance anti-inflammatory factors, and create a favorable immune microenvironment conducive to tissue repair and regeneration (34).

Introduction of stem cells into a host can significantly reduce the risk of graft rejection by modulating the immune response. This immunomodulatory effect is particularly important in regenerative medicine, where the success of stem cell therapies often depends on the acceptance of the transplanted cells by the host immune system. Stem cells establish an immunosuppressive microenvironment that aids engraftment and enhances tissue repair, which is crucial for the integration of transplanted cells into host tissue (35).

Moreover, the immunoregulatory functions of stem cells extend beyond the simple suppression of immune responses; they actively promote tissue repair and regeneration. This regenerative ability is frequently linked to immunomodulatory effects, because the repair process can trigger inflammatory responses that must be precisely controlled to prevent chronic inflammation and fibrosis. Stem cells play a critical role in healing after injury or transplantation by balancing immune responses and promoting tissue repair (36).

In summary, stem cell-mediated immune regulatory pathways encompass a complex interplay between cytokine secretion, signaling pathway modulation, and active promotion of tissue repair.

### 2.7 Crosstalk regulation of signaling pathways in stem cell therapy (**Figure 4**)

**Figure 4:**
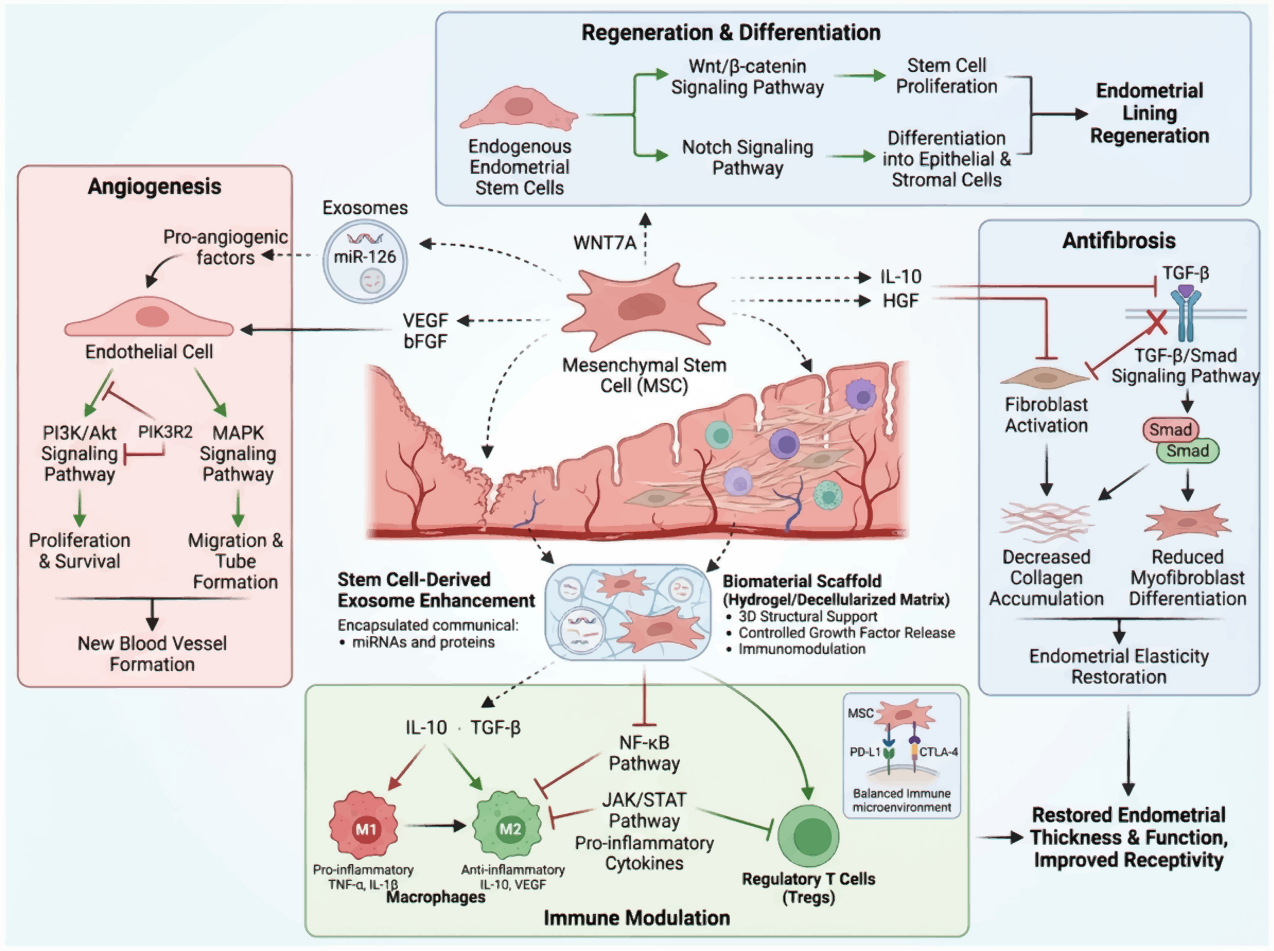
A conceptual signaling network diagram. Created by Biorender https://BioRender.com/h2idihg.

The intricate interplay between multiple signaling pathways forms a complex network that is crucial for the regulation of angiogenesis, fibrosis suppression, and immune modulation in stem cell therapy. Recent studies highlight a complex interplay between key developmental pathways (TGF-β, Wnt, Notch) and immune checkpoint signaling, informing novel combination therapies. Integrated computational analyses across cancer types reveal that the activity of the TGF-β, Wnt, and Notch pathways is inversely correlated with markers of antitumor immunity and favorable responses to anti-PD-1/PD-L1/CTLA-4 immunotherapy (37). This suggests that these oncogenic pathways can establish an immunosuppressive tumor microenvironment. Mechanistically, evidence indicates that pathways like Wnt/β-catenin may drive resistance to checkpoint inhibitors (38). Therefore, co-targeting these developmental pathways with immune checkpoints presents a compelling therapeutic rationale. Thise strategy can simultaneously disrupt pro-tumorigenic signaling, including the maintenance of cancer stem cells that are critically regulated by these pathways (39), and reverse immune suppression. Several small-molecule inhibitors targeting these pathways are in preclinical and early clinical development (40). Potential resistance mechanisms may include the compensatory activation of parallel or synergistic pathways, such as the Hippo pathway, which interacts with many developmental pathways (41). Tumors may also evolve cell-intrinsic escape mechanisms. This systems-level understanding directly translates into clinical trial design, advocating combination therapies that target the crosstalk between tumor-intrinsic oncogenic drivers and the immune system to overcome resistance and improve outcomes.

In summary, the regulation of signaling pathway crosstalk in stem cell therapy is a crucial research area with significant potential to advance regenerative medicine. Researchers can optimize stem cell therapies by unraveling the complex interactions between these pathways, ensuring that they are not only effective, but also safe for patients. This knowledge is crucial for the advancement of targeted therapies that fully utilize stem cells to treat various diseases and injuries. Continued research will provide new insights into the molecular mechanisms that regulate stem cell behavior and interactions with the immune microenvironment, leading to novel therapeutic approaches (42).

### 2.8 Integrated mechanistic framework

Based on the above review, we propose an integrated mechanistic framework linking molecular signaling, immunomodulation, and biomaterial strategies. TE is characterized by dysregulated molecular signaling, such as impaired proregenerative pathways and a profibrotic, immune-activated microenvironment that hampers repair. Stem cells, particularly MSCs and their exosomes, act as core regenerative agents by modulating this niche, promoting angiogenesis, reducing inflammation, and differentiating into endometrial cells. However, their efficacy is limited by poor survival in the hostile TE environment. Advanced biomaterial strategies are designed to address these limitations. Biomaterial scaffolds or hydrogels protect transplanted cells, improve cell retention, and can be engineered to actively remodel the niche. These strategies include delivering sustained growth factors, providing antioxidant support to counteract oxidative stress, offering immunomodulatory cues to suppress inflammation, and promoting regenerative immune responses. Thus, the framework integrates intelligent biomaterial design to shield and augment stem cells while directly correcting the dysregulated signaling and immune landscape of TE, offering a synergistic strategy for endometrial regeneration.

## 3 The role of the immune microenvironment in TE repair

### 3.1 Composition and function of immune cells in the endometrium

The endometrium is a dynamic tissue that houses a diverse array of immune cells including macrophages, dendritic cells, natural killer (NK) cells, and regulatory T cells (Tregs). These cellular components are essential for preserving tissue homeostasis and facilitating the reproductive processes. Macrophages are instrumental in tissue remodeling and clearance of apoptotic cells. Dendritic cells, on the other hand, serve as antigen-presenting cells that are critical for initiating adaptive immune responses. NK cells are particularly important in the context of early pregnancy, where they contribute to trophoblast invasion and the establishment of maternal-fetal immune tolerance. Endometrial natural killer (eNK) cells, also termed uterine NK (uNK) cells, exhibit distinct phenotypic and functional characteristics compared with their peripheral blood counterparts and play indispensable roles during implantation. Transcriptomic analyses reveal a coordinated upregulation of immune-related genes, including those stimulating uNK cell proliferation, such as interleukin-15 (IL-15) and its receptor IL-15Rα, within the secretory-phase endometrium, highlighting their cyclical dynamics (43). Functionally, eNK cells play a pivotal role in establishing a receptive environment and promoting extravillous trophoblast (EVT) invasion (44). Furthermore, their activity is closely linked to immune tolerance, involving mechanisms such as the expression of indoleamine 2, 3-dioxygenase (IDO), which inhibits T cell growth, and pathways that dampen cytolytic potential (45). However, the maternal hormonal environment profoundly influences eNK cells. Superovulation with gonadotropins, which is common in assisted reproductive technology (ART), leads to a significant reduction in endometrial CD56+ uNK cell density and impairs their proinvasive capacity towards EVTs (46). This alteration provides mechanistic insights into how controlled ovarian stimulation may contribute to suboptimal implantation and disorders of placentation (47). In summary, eNK cells are uniquely regulated, cycle-dependent lymphocytes essential for angiogenesis, trophoblast invasion, and maternal-fetal immune tolerance, and their number and function are susceptible to disruption by exogenous hormonal stimulation. A balanced and functional immune cell population is crucial to maintain a healthy endometrial environment that supports successful implantation and pregnancy.

In TE, compromised endometrial receptivity is a critical factor leading to implantation failure, with dysregulation of the local immune milieu playing a central role (48). This immune dysfunction manifests as altered profiles of uterine natural killer (eNK) cells and macrophages, which are essential for establishing immune tolerance and facilitating trophoblast invasion (49). Specifically, an imbalance towards a proinflammatory state, characterized by shifts in eNK cell cytotoxicity and a disrupted M1/M2 macrophage ratio, contributes to a hostile endometrial environment (50). This aberrant immune activation directly impairs decidualization, which is critical for embryo support. Increased expression of inflammatory factors such as allograft inflammatory factor-1 (AIF-1) in stromal cells inhibits their proliferation and decidualization capacity, thereby reducing receptivity (51). Furthermore, defective remodeling of the ECM, which is regulated by immune signaling and essential for trophoblast migration, occurs under such inflammatory conditions. The resulting chronic inflammation and inadequate decidual response create a suboptimal interface for embryo attachment and placental development (52). Consequently, these interconnected pathologies, defective decidualization, hindered trophoblast invasion, and sustained inflammation, synergistically lead to implantation failure and compromised endometrial receptivity in TE (53).

TE is characterized by a distinct and dysregulated immune microenvironment compared with the normal receptive endometrium. Emerging evidence points to a state of abnormal immune regulation in TE. Transcriptomic analyses have revealed the overexpression of specific genes, such as ribosomal protein L7 (RPL7) and RCC1 domain-containing 1 (RCCD1), which are implicated in and associated with aberrant immune infiltration patterns (54). This finding suggests a fundamental shift in the local immune landscape. Furthermore, critical changes were observed at the epithelial surface, which is the primary site of embryo attachment. The apical glycocalyx of luminal epithelial cells in TE shows significantly reduced expression of MECA-79 glycans (55). As these glycans serve as ligands for L-selectin on trophoblast cells, their deficiency is likely to impair the initial adhesive interactions crucial for implantation, indicating a compromised immunotolerant interface (56). While the specific proinflammatory cytokines (e.g., TGF-β, IL-1β) or tolerogenic factors (e.g., IL-10) were not directly measured in these studies, the identified molecular alterations collectively describe an immune milieu that is both dysregulated and less receptive. The proinflammatory shift and decrease in key adhesion molecules create a suboptimal environment for embryo implantation, contributing to the poor reproductive outcomes associated with TE.

### 3.2 Immune mechanisms in stem cell-mediated thin endometrial regeneration

As summarized above, MSC-based therapies have emerged as a promising therapeutic strategy for endometrial repair. A growing body of evidence suggests that the therapeutic efficacy of MSCs is largely mediated by immunomodulation rather than direct differentiation, thereby establishing a coherent mechanistic framework for endometrial regeneration.

Central to this mechanism is the profound modulation of the local immune microenvironment. MSCs, whether delivered exogenously or recruited endogenously via biomaterial scaffolds, exert potent immunomodulatory effects. A key action is the polarization of macrophages from a pro-inflammatory (M1) to an anti-inflammatory, pro-regenerative (M2) phenotype (57). This shift is crucial for reducing fibrosis and creating a favorable environment for repair. MSCs achieve this through their secretome, which includes a diverse array of cytokines, growth factors, and extracellular vesicles such as exosomes. Notably, even MSC-derived apoptotic bodies have been shown to induce macrophage immunomodulation and promote healing (58).

These immunomodulatory functions extend beyond macrophages. MSCs can inhibit lymphocyte activation and proliferation, and promote the differentiation of regulatory T and B cells, further dampening detrimental inflammatory responses. This coordinated immunomodulation reduces the expression of pro-inflammatory cytokines (e.g., IL-1β, TNF-α) while enhancing anti-inflammatory and pro-angiogenic mediators. Consequently, this altered microenvironment inhibits pathological fibrosis, promotes angiogenesis, and supports the proliferation of endometrial cells, leading to increased endometrial thickness, improved glandular development, and ultimately, the restoration of fertility (59). Thus, the primary mechanism for MSC-mediated regeneration in TE is the targeted reprogramming of the immune landscape to enable tissue repair and restore receptivity.

### 3.3 The interaction between the immune microenvironment and angiogenesis

Interaction between the immune microenvironment and angiogenesis is vital for endometrial regeneration, particularly in the context of stem cell therapy for TE. VEGF stimulates the development of new blood vessels, thereby enhancing blood perfusion and nutrient transport in regenerating endometrial tissue. This process is especially critical because a well-developed vascular network is indispensable for the viability and functionality of endometrial stem cells after transplantation, as well as for overall recovery following endometrial injury or surgical procedures (60). Additionally, cytokines and growth factors released by immune cells promote angiogenesis and modulate the local immune milieu, thereby creating conditions conducive to tissue regeneration. Macrophages can polarize into either proinflammatory M1 or anti-inflammatory M2 phenotypes, each of which differentially influences angiogenesis and immune dynamics in the endometrial microenvironment (61). The balance between these immune cell types is critical, because an excess of M2 macrophages can lead to immunosuppression, potentially hindering the effectiveness of stem cell therapies aimed at endometrial regeneration (62).

Stem cells significantly influence the immune microenvironment and indirectly enhance angiogenesis. MSCs possess immunomodulatory capabilities that affect the behavior of nearby immune cells. They secrete various factors that can shift the immune response towards a more regenerative phenotype, enhancing angiogenesis and tissue repair. For example, MSCs can produce exosomes rich in miRNAs and proteins that regulate the function of immune cells, thus promoting an environment that favors angiogenesis and inhibits excessive inflammation (63). This interaction highlights the importance of stem cells not only as potential sources for tissue regeneration but also as active participants in shaping the immune landscape, which is essential for successful angiogenesis and endometrial healing. For instance, studies have shown that an imbalance in the immune microenvironment, characterized by either excessive inflammation or immunosuppression, can adversely affect angiogenesis, leading to suboptimal outcomes in endometrial repair therapies (64).

In conclusion, the interaction between immune microenvironment and angiogenesis is a critical determinant of endometrial regeneration. Immune cells promote angiogenesis by secreting essential factors, whereas stem cells adjust their immune responses to foster an environment conducive to tissue repair. The coordination of these processes is crucial for restoring endometrial function and enhancing fertility. Future studies should clarify the mechanisms underlying these interactions to enhance stem cell therapies for endometrial regeneration and address issues related to TE.

### 3.4 Standardization challenges in stem cell transplantation

Stem cell therapy holds great promise for treatment of TE by regenerating damaged tissue. However, translating this promise into standardized clinical practice faces significant challenges, primarily due to the lack of consensus on key treatment parameters.

First, the optimal cell source remains unknown. Although various MSCs, including BMSCs, UC-MSCs, adipose-derived MSCs, endometrial MSCs (enMSCs), and menstrual-blood-derived MSCs (MenSCs), have shown efficacy (65), their comparative effectiveness is unclear. Furthermore, strategies to enhance cell function, such as the decidualization of MenSCs or niche remodeling of eSSCs (66), add another layer of complexity to standardization.

Second, administration routes and delivery methods vary widely, with no clearly superior approach. Cells are delivered via intrauterine infusion, transmyometrial/subendometrial injection, or scaffold-assisted methods using hydrogels (67). These methods significantly impact cell retention and engraftment; for instance, encapsulating stem cells in hydrogels, such as hyaluronic acid or collagen, prolongs their presence and improves therapeutic outcomes compared to cell suspension alone (68). The choice between local and systemic administration remains unclear due to a lack of definitive guidance.

Third, critical treatment protocols, including dosage, timing, and frequency of administration, are highly heterogeneous across studies (69). This variability, compounded by small sample sizes in most clinical trials, makes it difficult to establish a reproducible and effective regimen (70).

In conclusion, although stem cell therapy for TE is safe and shows encouraging results, its clinical integration is hampered by non-standardized practices regarding cell source, delivery, and dosage. Large-scale randomized controlled trials are needed to optimize these protocols and validate their long-term efficacy.

### 3.5 The role of biomaterials in modulating the immune microenvironment

Biomaterials play a crucial role in regenerative medicine, particularly in modulating the immune microenvironment. As carriers of stem cells, biomaterials can significantly influence local immune responses, promote immune tolerance, and enhance therapeutic outcomes. For instance, biomaterials can be engineered to release specific immunomodulatory factors that create a favorable environment for stem cell engraftment and function. This role is particularly important in contexts, such as TE regeneration, in which the local immune response can either support or hinder healing. Researchers can enhance tissue repair and regeneration using biomaterials that replicate the ECM, thereby optimizing interactions between stem and immune cells (71).

Biomaterials can be engineered to release cytokines or growth factors that attract anti-inflammatory immune cells and suppress proinflammatory responses. This dual mechanism not only mitigates inflammatory responses but also amplifies the regenerative capabilities of the delivered stem cells. Studies have demonstrated that such biomaterials improve outcomes in models of endometrial damage by establishing a supportive microenvironment that promotes cell viability and activity (72).

Advanced technologies such as three-dimensional (3D) bioprinting and decellularized matrix techniques have further enhanced the immunocompatibility of biomaterials. These innovations enable the formation of intricate tissue structures that mimic native tissue architecture, thereby enhancing integration and functionality after implantation. 3D bioprinting can create scaffolds that offer structural support and enable the spatial arrangement of stem and immune cells, thereby replicating the natural cellular environment. This spatial arrangement is critical for proper signaling and intercellular interactions, which are essential for effective tissue regeneration (73). In addition, decellularized matrices derived from native tissues retain bioactive molecules that can further modulate the immune response, thereby enhancing the compatibility of the implanted biomaterial with the host immune system.

Biomaterials play vital and complex roles in immune microenvironments. These materials act as carriers for stem cells, not only supporting cell survival and function but also actively engaging with the immune system to promote healing and tissue regeneration. Advancements in biomaterial engineering, such as 3D printed constructs and decellularized matrices, have shown significant potential for enhancing stem cell-based therapies in regenerative medicine, especially in complex scenarios such as endometrial repair (74). As research continues to elucidate the complex interactions between biomaterials, stem cells, and the immune microenvironment, significant advancements in the clinical applications of these technologies are likely to occur.

### 3.6 The interaction between the immune microenvironment and fibrosis

In TE, the persistence of inflammatory stimuli can lead to a pathological state characterized by excessive fibrotic tissue deposition, ultimately compromising reproductive potential. Macrophages and T cells are crucial in this process as they release profibrotic cytokines and chemokines that stimulate fibroblast activation and collagen deposition. Activated macrophages secrete transforming TGF-β, that induces fibroblast differentiation into myofibroblasts, thereby intensifying the fibrotic response. This chronic inflammatory milieu not only fosters fibrosis but also creates a vicious cycle in which fibrotic tissue perpetuates further immune dysregulation, leading to a sustained inflammatory state that hinders effective tissue repair (75).

Stem cell therapy represents a promising therapeutic strategy for modulating the immune microenvironment and mitigating fibrosis in conditions such as TE. These cells exhibit distinctive immunomodulatory capabilities that enable them to reshape the local immune milieu through the downregulation of profibrotic mediator production and enhancement of anti-inflammatory cytokine secretion. For instance, MSCs secrete various soluble factors that inhibit macrophage activation and skew T cell responses towards a regulatory phenotype, thereby reducing the overall inflammatory burden. Stem cells support tissue regeneration by enhancing the survival and proliferation of the resident endometrial cells, which are essential for restoring normal endometrial function. Stem cells offer dual benefits in addressing both the fibrotic and inflammatory components of TE by dampening the immune response and facilitating tissue repair (76).

**Figure 5:**
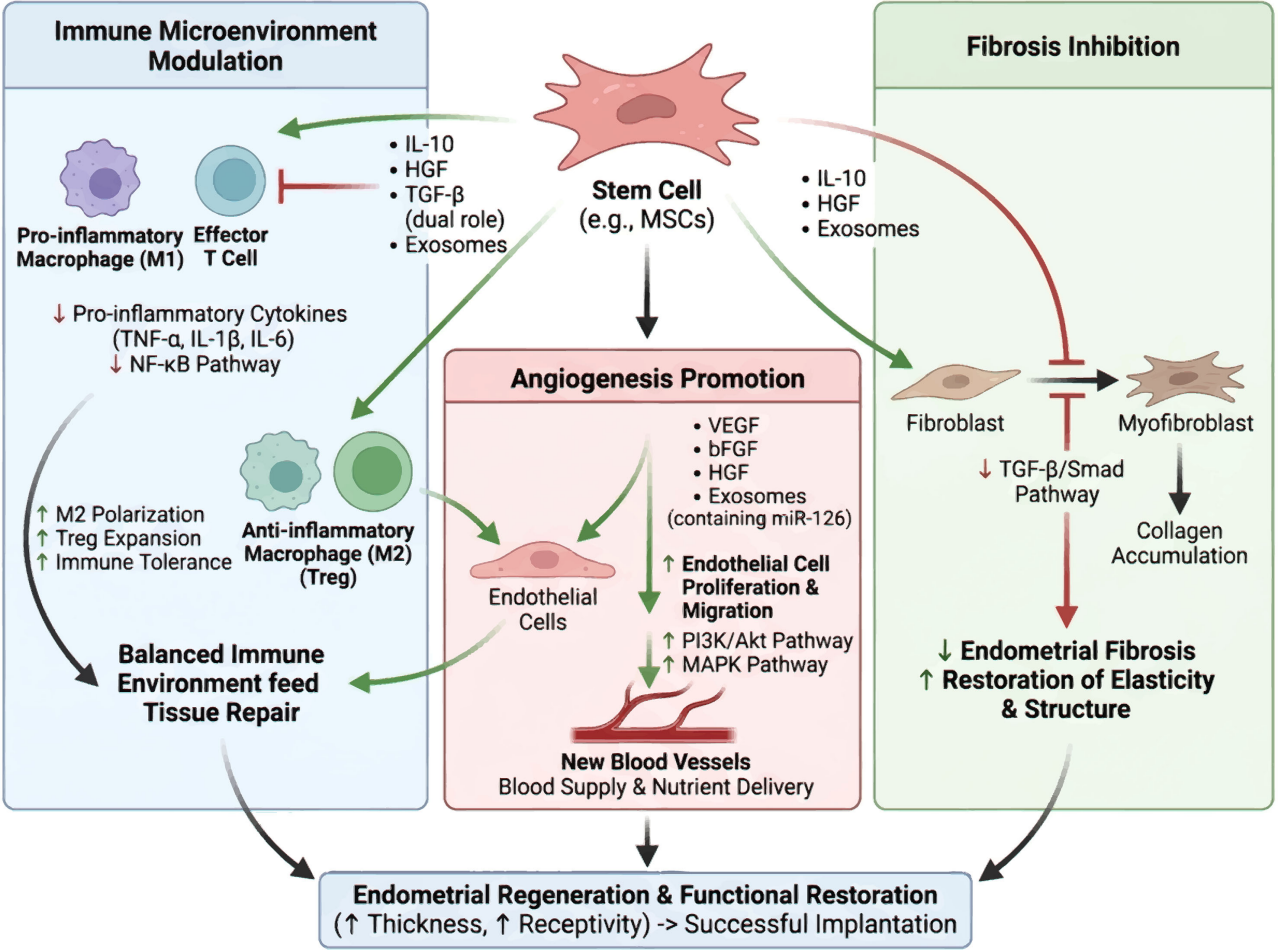
A conceptual illustration of immune-angiogenesis-fibrosis interactions. Created by Biorender https://BioRender.com/h2idihg.

Bidirectional regulation of the immune system and fibrosis represents a critical area of focus for the development of effective treatments for TE. Understanding the intricate interactions between immune cells and fibroblasts may reveal novel therapeutic targets to restore the balance between tissue repair and fibrosis. For example, targeting specific immune pathways that promote fibrosis could help mitigate excessive scarring, while simultaneously enhancing the regenerative potential of the endometrium. Furthermore, therapies that harness the immunomodulatory capabilities of stem cells could provide a multifaceted approach for treating TE by addressing existing fibrosis and preventing future fibrotic progression by maintaining a healthy immune microenvironment. This dual strategy underscores the significance of incorporating an immunological understanding into fibrotic condition management, thereby facilitating the development of novel therapeutic approaches (77). A conceptual illustration of the immune-angiogenesis-fibrosis interactions is provided in **Figure 5**.

### 3.7 Future research directions in immune microenvironment regulation

The immune microenvironment is crucial for endometrial repair and regeneration, especially under conditions such as TE, which greatly affects reproductive health. Understanding the specific roles of immune cell subsets in endometrial repair is essential to advance therapeutic approaches. Recent studies have indicated that immune cells, including macrophages, T cells, and dendritic cells, are actively involved in tissue remodeling and healing processes. Macrophages can adopt either pro- or anti-inflammatory states, which influence the balance between tissue injury and recovery (78). Exploring the detailed mechanisms by which these immune cells interact with stem cells and the extracellular matrix during endometrial regeneration may reveal novel therapeutic opportunities. In addition, understanding the signaling pathways and molecular signals that control immune cell recruitment and activation is critical for designing strategies to enhance the regenerative capacity of stem cell-based treatments. This study highlights the importance of a comprehensive understanding of the immune environment in the endometrium to develop more effective treatments for TE-related infertility.

Exploring precise immunomodulation strategies is crucial for improving the efficacy and safety of stem cell therapies in treating TE, while also clarifying the roles of immune cells. The current approaches often lack specificity and may inadvertently induce adverse immune responses, thereby undermining the therapeutic benefits of stem cell interventions. Precision immunotherapy aims to tailor immune modulation to the needs of individual patients and potentially improve outcomes. Employing biomaterials capable of the controlled release of immunomodulatory factors can enhance the immune environment, thereby supporting stem cell engraftment and function (79). Moreover, combining stem cell therapy with pharmacological agents that target specific immune pathways may synergistically enhance endometrial repair. Future research should aim to develop and validate precise immunomodulation strategies and evaluate their effects on the safety and regenerative efficacy of stem cell treatments. This approach shows the potential for addressing immune rejection challenges and enhancing the therapeutic efficacy of stem cells in reproductive medicine.

Integrating advanced technologies, such as single-cell sequencing and spatial omics, will improve our understanding of the dynamic changes in the immune microenvironment during endometrial repair. These cutting-edge techniques allow for the detailed characterization of immune cell populations and their interactions with the endometrial tissue at an unprecedented resolution. Researchers can identify specific gene expression profiles associated with different immune subtypes and their functional states in the context of TE by using single-cell RNA sequencing (80). On the other hand, spatial omics enables the mapping of immune cell distribution and activity within the endometrial tissue architecture, providing insights into how spatial relationships influence immune responses and tissue regeneration. The integration of these technologies will reveal not only the temporal dynamics of immune cell behavior but also the ways in which these changes correlate with the efficacy of stem cell therapies. Future research should utilize these novel methods to explore the intricacies of the immune microenvironment, thereby facilitating the development of more precise and effective regenerative treatments for women with conditions, such as TE. This study highlights the importance of a multidisciplinary approach that integrates immunology, stem cell biology, and advanced genomic technologies to address pressing challenges in reproductive health.

## 4 Combined application of biomaterials and stem cells to promote endometrial regeneration

### 4.1 Advantages of biomaterials as carriers for stem cells

Biomaterials are essential in regenerative medicine, particularly in stem cell-based treatments. A key benefit of biomaterials is their capacity to create 3D scaffolds that accurately replicate natural tissue environments, such as the endometrium. These structures enhance stem cell attachment and viability, both critical for effective tissue repair. The 3D structure of biomaterials enhances the interactions between cells and the extracellular matrix, thereby facilitating stem cell proliferation and differentiation into specific lineages. Research indicates that hydrogels and biomaterials support stem cell properties and improve their functionality by providing essential biochemical and mechanical signals for cell behavior (81). The incorporation of bioactive factors into these biomaterials can enhance stem cell activity, thereby improving tissue repair and regeneration outcomes (82).

Another significant advantage of biomaterials is their controlled-release functionality, which allows for the sustained delivery of growth factors and cytokines. This controlled release mechanism is crucial for improving stem cell therapy outcomes by providing bioactive molecules to cells over time, thereby replicating natural healing processes. Hydrogels can be engineered to release growth factors in a controlled spatial and temporal manner, enhancing the effectiveness of stem cell therapies in clinical applications, such as wound healing and tissue regeneration (83). This sustained release can enhance angiogenesis, collagen synthesis, and overall tissue repair, all of which are critical for successful outcomes in regenerative medicine (84). Customization of the release profiles of these factors to meet tissue-specific requirements represents a major advancement in this field.

Moreover, the mechanical properties of biomaterials significantly affect the stem cell behavior and differentiation pathways. Biomaterials can be engineered to elicit specific cellular responses by adjusting their stiffness, elasticity, and porosity. Research has indicated that the mechanical properties of hydrogels influence stem cell differentiation, directing them towards osteogenic or chondrogenic pathways based on substrate stiffness (85). The manipulation of biomaterial properties to influence stem cell fate is a powerful tool in tissue engineering, enabling the tailoring of therapeutic strategies for various clinical needs.

In summary, biomaterials offer several advantages as carriers for stem cells, including providing a supportive 3D microenvironment, enabling the controlled release of bioactive factors, and influencing stem cell behavior through mechanical properties.

### 4.2 Application of decellularized uterine matrix scaffolds

Decellularized uterine matrix scaffolds represent a notable advancement in regenerative medicine, especially for the treatment of uterine factor infertility. Scaffolds are produced through a precise decellularization process that eliminates cellular components while preserving the structural integrity and composition of the extracellular matrix. Preservation of extracellular matrix structure and composition is vital for reducing the risk of immune rejection, which is a frequent issue in tissue engineering. Research indicates that hydrostatic pressure methods for decellularization preserve structural proteins and mechanical properties more effectively than traditional detergent-based techniques (86). Decellularized scaffolds maintain the natural extracellular matrix architecture, thereby providing a supportive environment that encourages cell adhesion, migration, and proliferation, which are essential for successful tissue regeneration. Decellularized scaffolds can be functionalized with bioactive molecules to improve their regenerative capabilities, thereby providing a promising solution for the reconstruction of damaged uterine tissue (87).

The ability of decellularized scaffolds to promote stem cell colonization is another important aspect of their application. These scaffolds provide a niche for stem cells, thereby aiding their engraftment and differentiation into uterine-specific cells. Research indicates that stem cells seeded onto decellularized uterine scaffolds exhibit enhanced viability and functionality, contributing to the reconstruction of a functional endometrium (88). The scaffolds not only provide a physical structure, but also deliver biochemical signals that can guide stem cell behavior, thereby supporting the complex processes involved in endometrial regeneration. Scaffold-stem cell interactions are essential for functional outcomes, such as restoring menstrual cycles and enhancing fertility rates in animal models (89).

Preclinical studies have highlighted the biocompatibility and regenerative efficacy of decellularized uterine scaffolds, indicating their potential for clinical use. For example, *in vivo* studies have shown that these scaffolds can successfully integrate into the host tissue, promote angiogenesis, and reduce fibrosis (90). These scaffolds have demonstrated support for uterine tissue regeneration in animal models, resulting in enhanced reproductive outcomes such as successful pregnancies. The ability to create a vascularized and functional uterine environment is essential for the success of any tissue-engineered solution aimed at treating uterine factor infertility. These studies underscore the potential of decellularized scaffolds in reconstructive gynecology and suggest avenues for future clinical trials and therapeutic applications in humans (91).

In conclusion, decellularized uterine matrix scaffolds demonstrate significant potential for advancing regenerative medicine and addressing the challenges associated with uterine factor infertility. These scaffolds support tissue regeneration by maintaining the natural ECM structure and promoting stem cell colonization. The promising outcomes of preclinical studies highlight the necessity for continued research and clinical investigations to effectively utilize decellularized scaffolds to restore uterine function and enhance reproductive outcomes in infertile women. Advancements in optimizing decellularization protocols and comprehending immunological interactions are essential for improving the efficacy and safety of innovative therapeutic strategies (92).

### 4.3 Utilization of 3D bioprinting for endometrial repair

3D bioprinting technology has transformed tissue engineering, particularly in the context of endometrial repair. This innovative approach allows the precise construction of biologically functional endometrial tissue models that closely mimic the natural architecture and cellular composition of the human endometrium. Researchers can design scaffolds using bioinks composed of diverse biomaterials and cell types that provide structural support and promote biochemical interactions essential for tissue regeneration. A previous study demonstrated that a bilayer endometrial construct composed of a sodium alginate-hyaluronic acid hydrogel successfully restored the morphology and function of the endometrial wall in a rat model with partial uterine excision (93). Such constructs can accurately replicate the upper layer of endometrial epithelial cells and lower layer of stromal cells, demonstrating the potential of 3D bioprinting to create complex tissue architectures that support normal endometrial function.

Moreover, 3D bioprinting supports multicellular coculture systems, which are crucial for promoting cell-to-cell interactions and tissue formation. This capability is crucial for endometrial repair, in which the interactions between epithelial, stromal, and endothelial cells are fundamental for effective regeneration. 3D bioprinting improves the physiological relevance of the fabricated tissues through the concurrent deposition of multiple cell types. Research has demonstrated that a hydrogel scaffold incorporating human induced pluripotent MSCs significantly increases the viability and functionality of transplanted cells, thereby promoting endometrial regeneration (94). Establishing a supportive *in vitro* environment for these cells enhances their therapeutic effectiveness and facilitates the investigation of the complex cellular dynamics in endometrial regeneration.

In addition to its applications in tissue modeling and co-culture systems, 3D bioprinting offers a versatile platform for personalized treatment strategies and drug screening. Researchers can evaluate the efficacy of therapeutic interventions tailored to individual patient requirements by creating patient-specific endometrial models. This personalized approach is particularly valuable in cases of severe endometrial damage in which traditional treatments may fall short. The combination of sustained-release growth factor systems with 3D-printed scaffolds has demonstrated the potential to enhance endometrial regeneration and reproductive outcomes in preclinical models (95). These developments highlight the ability of 3D bioprinting to facilitate tissue repair and serve as a significant tool for drug discovery and testing in reproductive medicine.

The use of 3D bioprinting technology for endometrial repair represents a major advancement in regenerative medicine. Three-dimensional (3D) bioprinting has the potential to transform the management of endometrial injuries and related fertility issues through precise construction of functional tissue models, promotion of multicellular interactions, and facilitation of personalized therapeutic strategies. Ongoing research is expected to yield innovations that will improve the efficacy and safety of endometrial repair strategies, thereby enhancing reproductive health outcomes in women with endometrial dysfunction.

### 4.4 Nanocarriers and drug delivery systems

Nanocarriers represent a transformative method of drug delivery that significantly improves the targeted transport of bioactive molecules. Nanoscale vehicles such as liposomes, polymeric nanoparticles, and inorganic nanoparticles enhance drug bioavailability and therapeutic efficacy by promoting targeted accumulation and reducing systemic exposure. Nanocarriers possess distinct physicochemical properties, including size, shape, and surface charge, that enable them to traverse biological barriers more efficiently than conventional drug formulations. The enhanced permeability and retention (EPR) effect enables nanocarriers to preferentially accumulate in tumor tissues, leading to higher local drug concentrations and improved therapeutic outcomes (96). Additionally, modifying nanocarrier surfaces with targeting ligands can improve the specificity for diseased tissues, such as inflamed or cancerous regions, thereby enhancing drug delivery efficiency and minimizing off-target effects.

The combination of nanocarrier systems and stem cell therapy offers a synergistic strategy to enhance endometrial regeneration and immune regulation. MSCs have significant potential in regenerative medicine because of their ability to differentiate into various cell lineages, release bioactive molecules that support tissue healing, and regulate immune functions (97). When combined with nanocarriers, these stem cells can deliver therapeutic agents more effectively to the target site, enhancing the regenerative processes within the endometrium. MSCs encapsulated in nanocarriers can be modified to release growth factors or anti-inflammatory cytokines, thereby promoting endometrial regeneration and modulating the immune microenvironment (98). This combined method enhances treatment efficacy and enables controlled release of the therapeutic agent, thereby reducing the potential side effects of systemic administration.

Furthermore, nanocarriers can significantly reduce the adverse effects commonly associated with traditional drug therapies. These systems enhance treatment safety through targeted delivery, thereby reducing the exposure of healthy tissue to cytotoxic agents. For instance, conventional chemotherapeutics often cause severe side effects because of their lack of specificity. Nanocarriers encapsulate drugs and release them in a controlled manner at the tumor site, thereby reducing systemic toxicity (99). Recent progress in smart nanocarrier design, which responds to tumor microenvironment stimuli such as pH or enzyme activity, has improved drug delivery precision by releasing therapeutic agents at the required time and location (100). This targeted approach not only improves patient outcomes but also contributes to an improved quality of life during treatment.

In summary, integration of nanocarriers into drug delivery systems represents a notable advancement in regenerative medicine and cancer therapies. Nanocarriers enhance the therapeutic efficacy and patient safety by improving the targeted delivery of bioactive molecules, promoting synergy with stem cell therapies, and minimizing the side effects of conventional treatments. As research continues, the potential of these innovative systems to transform clinical practice and enhance treatment outcomes remains substantial (101).

### 4.5 Mechanisms of biomaterials regulating the immune microenvironment

Modulation of the immune microenvironment using biomaterials is a promising approach for improving therapeutic outcomes, especially in tissue engineering and regenerative medicine. Biomaterials predominantly influence therapeutic outcomes. Researchers can guide macrophage behavior by facilitating a shift from the proinflammatory M1 state to the tissue-regenerative M2 phenotype by engineering biomaterials with specific surface characteristics and bioactive elements. Additionally, certain biomaterials can enhance the production of anti-inflammatory cytokines, such as IL-10, while reducing levels of pro-inflammatory mediators like TNF-α and IL-6, thereby establishing a local immune milieu that supports healing processes (102). This immunomodulatory regulatory mechanism is critical for improving implant integration and for lowering the likelihood of chronic inflammation and fibrosis, which are common obstacles to effective tissue repair. Biomaterials not only affect macrophage polarization but also enhance immune tolerance and decrease the risk of transplant rejection. This immunomodulatory effect is crucial for stem cell therapies and organ transplants because the immune response can greatly affect the success of these interventions. Biomaterials can replicate the ECM and deliver signals that encourage the development of Tregs, which are crucial for sustaining the immune balance and curbing excessive immune reactions. For example, the incorporation of immunomodulatory factors or the use of materials that release bioactive molecules in a controlled manner can enhance the local production of Tregs, thereby fostering an environment that supports the acceptance of transplanted cells or tissues (103). This approach not only aids in minimizing rejection but also enhances the overall efficacy of regenerative therapies.

Furthermore, the design of smart responsive materials that can dynamically modulate the immune microenvironment represents a significant advancement in biomaterial science. These materials can adjust their immunomodulatory properties in real time by responding to physiological triggers such as pH changes, temperature variations, or specific biomolecules. Materials that release anti-inflammatory agents in response to inflammatory signals can mitigate excessive immune responses at sites of injury or implantation (104). These intelligent biomaterials enhance the therapeutic efficacy by optimizing the local immune environment and ensuring that the immune response aligns with the healing process. The integration of these advanced biomaterials into clinical practice could revolutionize the management of various conditions, particularly those involving complex immune interactions such as autoimmune diseases, chronic inflammatory conditions, and tissue injuries.

In summary, biomaterials influence the immune microenvironment through diverse mechanisms, including modulation of macrophage polarization, enhancement of immune tolerance, and creation of smart responsive materials. These strategies improve biomaterial integration and effectiveness in therapy while also introducing novel methods for addressing immune-related challenges in regenerative medicine. Ongoing research is expected to yield insights that will enhance the development of personalized treatment strategies utilizing the immune system for tissue repair and regeneration (105).

### 4.6 Advances in preclinical studies on stem cell-biomaterial combination therapy

Integrating stem cell therapy with biomaterials has shown promise in preclinical studies for improving endometrial regeneration, especially for treating TE and intrauterine adhesions (IUAs). Numerous animal model studies have demonstrated that this combined approach significantly promotes restoration of endometrial thickness and functionality. Research has indicated that combining MSCs with biomaterial scaffolds supports cell survival and delivers bioactive factors crucial for tissue repair (106). Biomaterials such as natural polymers, including collagen and hyaluronic acid, function as scaffolds that mimic the ECM, thereby facilitating cell adhesion, proliferation, and differentiation. Additionally, studies have reported that the combination of MSCs with biomaterials improves angiogenesis and reduces fibrosis, thereby addressing common challenges associated with endometrial injury (107).

Evaluation of the survival rate of transplanted cells, immune response, and overall tissue repair effects are critical components of preclinical studies. Injectable hydrogels containing stem cells demonstrated improved cell retention and viability at injury sites, which are essential for successful regeneration (108). Furthermore, when combined with biomaterials, the immunomodulatory properties to modulate the local immune environment, promoting favorable conditions for healing while minimizing adverse inflammatory responses (109). This combined approach enhances the therapeutic efficacy and facilitates a deeper understanding of the interactions between stem cells and their biomaterial carriers.

The theoretical and experimental foundations of these preclinical studies are vital for the eventual translation of these therapies into clinical settings. Understanding how stem cell-biomaterial combinations improve endometrial regeneration will enable the development of targeted and effective treatments for infertility caused by endometrial dysfunction. For instance, findings from various animal models have highlighted the importance of optimizing biomaterial properties, such as degradation rates and mechanical strength to align with the dynamic requirements of the regenerating endometrium (110). The encouraging outcomes of preclinical models justify progression to clinical trials to comprehensively assess the safety and efficacy of these combined therapies in humans.

In conclusion, progress in preclinical research on stem cell-biomaterial combination therapy underscores its potential as a transformative approach for addressing endometrial pathologies. Animal studies have revealed synergistic effects that advance our understanding of tissue regeneration and form the basis for innovative clinical strategies to restore endometrial function and improve reproductive outcomes. As research continues to evolve, the integration of advanced biomaterials with stem cell therapies holds great promise in overcoming the limitations of current treatment modalities for endometrial disorders (111).

### 4.7 Current status and challenges of clinical applications

**Figure 6:**
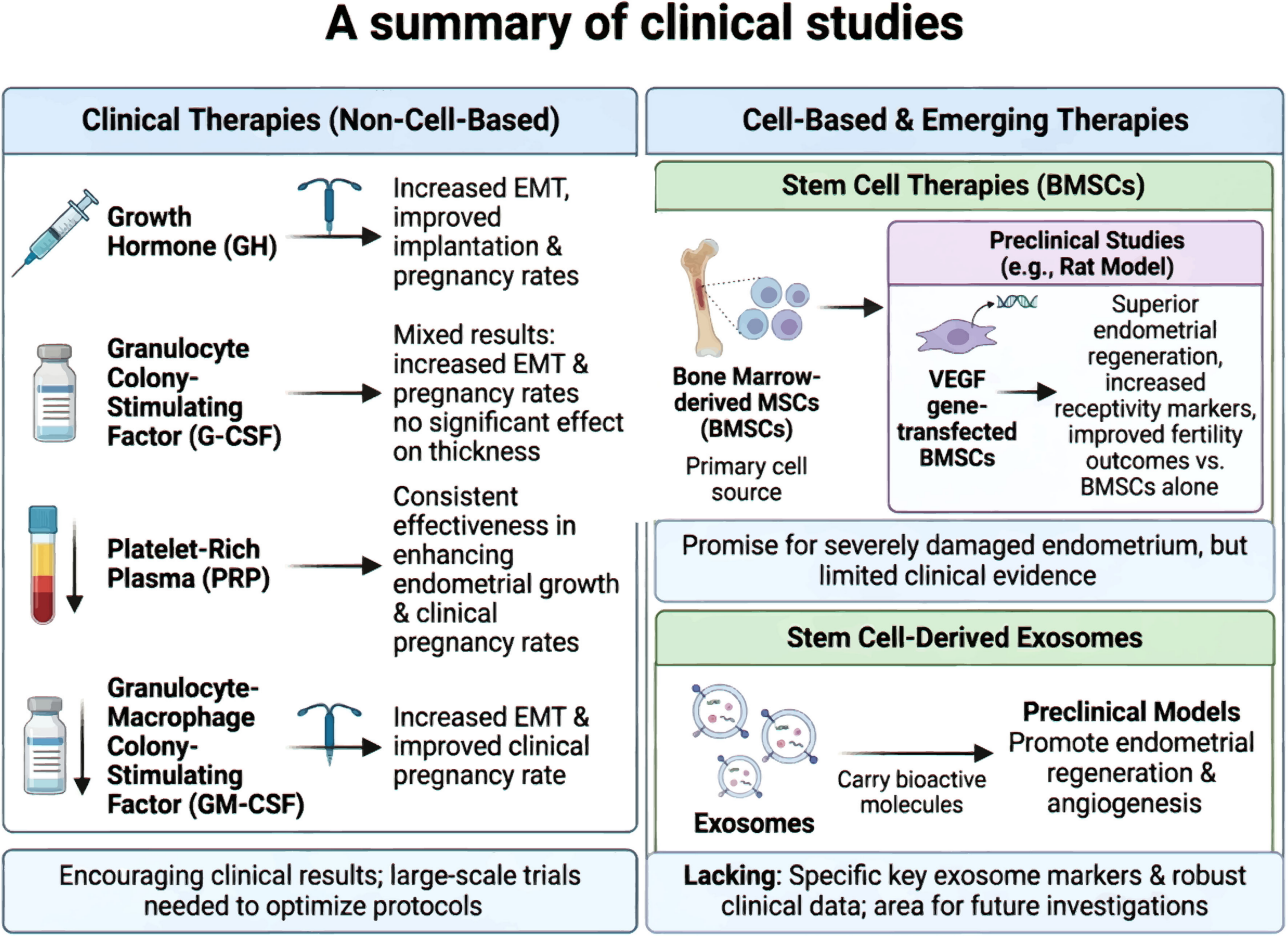
A summary of clinical studies. Created by Biorender https://BioRender.com/h2idihg.

Recent clinical studies have focused on various regenerative and immunomodulatory therapies to address this refractory condition (**Figure 6**).

Clinical trials have investigated the efficacy of growth hormone (GH), granulocyte colony-stimulating factor (G-CSF), platelet-rich plasma (PRP), and granulocyte-macrophage colony-stimulating factor (GM-CSF)in treating TE. Intrauterine administration of GH significantly increases epithelial-mesenchymal transition (EMT) and improves implantation and clinical pregnancy rates in women with refractory TE (112). Similarly, studies on G-CSF have shown mixed but promising results, with several reporting increased EMT and improved pregnancy rates (113). However, some trials did not find a significant effect on endometrial thickness (114). PRP infusion has consistently demonstrated effectiveness in enhancing endometrial growth and clinical pregnancy rates in frozen-thawed embryo transfer cycles (115). A randomized study further confirmed that intrauterine GM-CSF infusion effectively increases EMT and improves clinical pregnancy rate in patients with persistent TE (116).

BMSCs are a primary cell source for cell-based therapies. Preclinical studies using a rat model, have shown that transplantation of VEGF gene-transfected BMSCs promotes superior endometrial regeneration, increases receptivity markers, and improves fertility outcomes compared with BMSCs alone (117). Although stem cell therapies hold promise in restoring severely damaged endometrium (118), clinical evidence remains limited. Stem cell-derived exosomes have recently emerged as a novel therapeutic approach. These exosomes, which carry bioactive molecules, have demonstrated potential in preclinical models for promoting endometrial regeneration and angiogenesis (119). However, key exosome markers and robust clinical data for their use in TE treatment are still lacking, representing areas for future investigation (111). In summary, while regenerative therapies such as PRP, G-CSF, and GH show encouraging clinical results, further large-scale trials are needed to optimize protocols and validate the therapeutic potential of advanced approaches, such as stem cells and their exosomes (120).

Stem cell-based therapies, particularly those using MSCs, represent a promising avenue in TE treatment. However, their long-term safety profiles require rigorous evaluation. Key concerns include unintended biodistribution and low engraftment rates of transplanted cells, which may limit efficacy and pose unknown systemic risks (121). Upon repeated administration, immune reactions remain a concern, as evidenced by reported cases of urticaria in clinical trials (122). Although MSCs are generally considered to have low immunogenicity, the theoretical risk of tumorigenicity from residual cellular components or supporting biomaterials cannot be dismissed without additional data (123). Current clinical evidence, often from small-scale studies with short follow-up periods, indicates that these therapies are well tolerated in the near term (124). Nevertheless, there is a clear and pressing need for long-term follow-up in robust clinical trials to systematically monitor any delayed adverse effects, ensuring that the therapeutic benefits truly outweigh the potential risks.

Stem cell-derived exosome therapy for TE faces significant long-term safety concerns, primarily owing to the evolving regulatory framework. The product definition of exosomes is complex, as they are vesicles with diverse cargo, making the standardization of their therapeutic use challenging (125). Good Manufacturing Practice (GMP) compliance is required to ensure batch-to-batch consistency, purity, and safety, which is difficult given the intricate processes for isolating and characterizing exosomes (126). Furthermore, scaling up production for widespread clinical use presents major hurdles, as maintaining the biological activity and functional properties of exosomes during large-scale manufacturing remains a critical obstacle to clinical translation.

**Figure 7:**
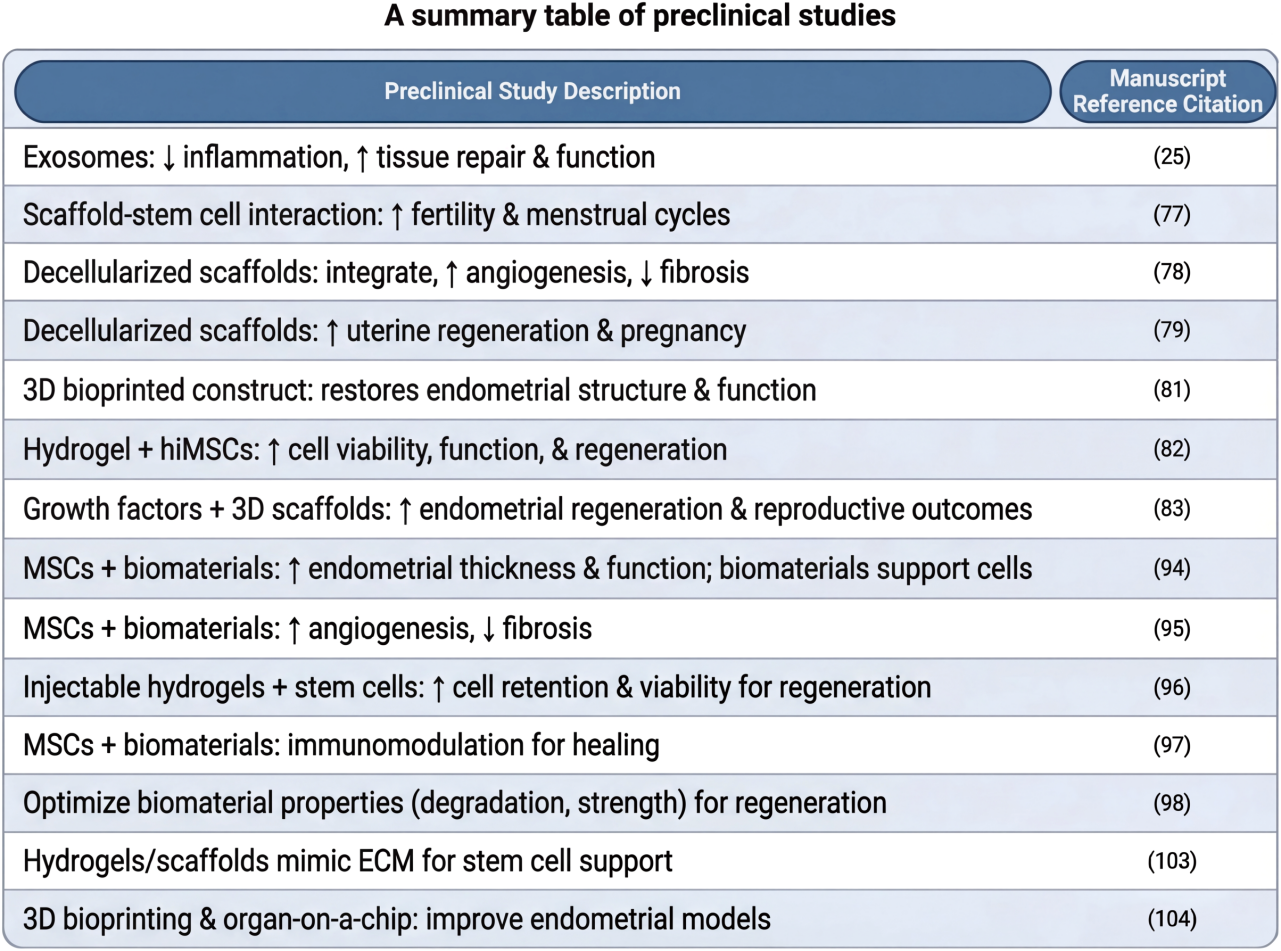
A summary table of preclinical studies. Created by Biorender https://BioRender.com/h2idihg.

Current evidence on stem cell therapy for TE, although promising, has several limitations in terms of scope and methodology. The dataset is primarily derived from specific stem cell sources, such as MSCs or BMSCs (127), which may not represent the full spectrum of potential therapeutic progenitor cells. This narrow focus limits the generalizability of these findings to other stem or progenitor cell populations (128). Furthermore, evaluation metrics often rely on *in vitro* assays (e.g., cell proliferation and cytokine levels) and preclinical animal models (129), introducing potential biases, as these may not fully correlate with *in vivo* endometrial regeneration and clinical pregnancy outcomes in humans. The generalizability of these preclinical findings to a heterogeneous population of patients with TE remains uncertain (130). Finally, the current evidence base lacks large-scale, long-term studies, making it difficult to comprehensively assess the durability of therapeutic effects and potential safety concerns. A summary table of the preclinical studies is provided in **Figure 7**.

## 5 Discussion

In summary, stem cell therapy for TE represents a promising advancement in reproductive medicine and is characterized by complex molecular and immunological processes that facilitate endometrial regeneration. The development of this field reflects a sophisticated understanding of how stem cells coordinate multiple signaling pathways to promote angiogenesis, inhibit fibrosis, and facilitate cellular proliferation and differentiation, which are essential for restoring endometrial structure and function.

Despite the array of therapeutic options explored for TE, significant knowledge gaps persist. The mechanisms underlying the actions of many promising treatments remain incompletely understood. A major translational challenge is the lack of standardized protocols, particularly for regenerative approaches such as PRP and stem cell therapies, with variations in preparation methods, administration timing, and dosage hindering the comparability of results and clinical application. Furthermore, the evidence base is constrained by methodological limitations in the study design, including small sample sizes, observational or pilot studies, and heterogeneous patient populations, which prevent definitive conclusions regarding efficacy and safety. For emerging frontiers such as stem cell-derived exosomes or advanced biomaterial-based drug delivery systems, compelling preclinical data exist; however, robust clinical evidence is still scarce and requires validation through large-scale, well-controlled trials. Finally, there is no clear consensus on the optimal treatment strategy or hierarchy, with network meta-analyses suggesting the potential superiority of certain immunomodulatory agents. Meanwhile, some studies present conflicting results on combination therapies, underscoring the need for more rigorous comparative effectiveness research.

Consequently, future research on TE treatment should adopt a more structured perspective and integrate molecular signaling, immunomodulation, and biomaterial strategies. The primary focus should be on elucidating and targeting specific molecular pathways that govern endometrial regeneration and fibrosis to develop precise therapeutic interventions. Concurrently, advancing immunomodulatory strategies is crucial because biomaterials and stem cell-derived components can regulate the local immune microenvironment to reduce fibrosis and promote repair. Future work should prioritize the development of advanced scaffolds, such as those derived from decellularized endometrial extracellular matrix or fabricated via 3D bioprinting, which more accurately mimic the biomechanical and biochemical properties of native tissue to support cellular engraftment and function. Furthermore, smart biomaterial systems capable of spatiotemporally controlling the delivery of stem cells, their extracellular vesicles, growth factors, and immunomodulatory agents to the injury site must be explored. Finally, the convergence of emerging technologies such as 4D printing, AI-assisted monitoring, and patient-specific organoid models with these core strategies is essential for creating personalized and effective therapies to restore endometrial function and fertility.

In summary, this review provides novel insights into treating TE by shifting the pathological understanding from cellular quantity reduction to functional impairment, as single-cell RNA sequencing reveals stromal progenitor dysfunction, impaired angiogenesis, immune dysregulation, and cellular senescence in TE. It introduces a therapeutic paradigm from exogenous cell transplantation to programmed control of endogenous MSCs through multifunctional biomaterials, such as in situ-forming hydrogels that enable “Recruit-Anchor-Differentiate” programming for targeted tissue regeneration. Allogeneic MSCs, particularly umbilical cord-derived, are emphasized as promising off-the-shelf therapies due to their regenerative and immunomodulatory effects for endometrial repair. The review further constructs an integrative conceptual framework of biomaterial-mediated programmed regeneration, where smart hydrogels provide spatiotemporal control to overcome stem cell recruitment, retention, and differentiation challenges. These perspectives collectively advance the field towards mechanism-targeted strategies that leverage omics insights and rational biomaterial design for next-generation, clinically translatable regenerative medicine.
